# Epigenetic regulation of drug metabolism in aging

**DOI:** 10.18632/aging.203312

**Published:** 2021-07-11

**Authors:** Joseph L. McClay

**Affiliations:** 1 Department of Pharmacotherapy and Outcomes Science, School of Pharmacy, Virginia Commonwealth University, Richmond, VA 23298, USA

**Keywords:** DNA methylation, histones, cytochrome P450, xenobiotic metabolism, liver

Adverse drug reactions (ADRs) occur more frequently as people live longer, have greater numbers of chronic conditions, and take more medications. Older adults aged 65+ are estimated to account for over one third of all emergency department visits for ADRs and have the highest rates of hospitalization for ADRs of any age group [1]. Moreover, it has been estimated that more than 80% of ADRs in older patients are dose related and therefore avoidable [2]. This implies that effective methods for predicting the correct dose for the individual patient would be transformational in geriatric healthcare. Among the factors known to influence drug response in aging include changes to organ system function and body composition. However, these aging effects are considered too coarse to guide dosing decisions and chronological age itself has repeatedly been shown to have limited predictive value [3]. Therefore, there is an unmet need for novel biomarkers to guide dosing decisions in geriatric healthcare.

When considering biomarkers of drug metabolism, the first examples that often spring to mind are cytochrome P450 genetic polymorphisms that predict drug metabolism phenotypes. For example, polymorphisms at the cytochrome P450 2D6 (*CYP2D6*) gene have been tremendously successful in predicting the large individual differences in activity of its encoded enzyme [4]. However, an individual’s genome sequence is largely stable across the life course except for random somatic mutations. Therefore, pharmacogenetic analysis of common DNA sequence variants has limited power to explain changes in drug response across the life course of the same individual. In recent years, however, the dramatic epigenetic changes that accompany aging have been recognized [5]. We and others have hypothesized that epigenetic changes in aging could affect the regulation of genes involved in drug metabolism [6].

Recently we reviewed prior human epigenome-wide studies of aging and identified cytochrome P450 2E1 as the drug metabolism gene showing most evidence for age-related epigenetic change^7^. Indeed, *CYP2E1* was one of the loci that comprised the DNA methylation clock of Horvath (2013) [5]. To determine if these epigenetic changes affected expression and metabolic activity of CYP2E1 in the liver, the primary organ of xenobiotic metabolism, we assayed mouse tissue from aged mice (4-32 months) sourced from the National Institute on Aging rodent tissue bank. A mouse model affords a greater experimental control than a human study and the age-associated differentially methylated region (a-DMR) found in humans had a clear-cut homolog in mice.

We first showed that DNA methylation levels at mouse *Cyp2e1* increased with age in liver, with a concomitant reduction in gene expression. Furthermore, histone H3 lysine 9 acetylation (H3K9ac) but not H3K27ac changed with age at the same locus. To test if these epigenetic changes with age correlated with CYP2E1 activity, we assayed the rate of metabolism of the CYP2E1-specific probe drug chlorzoxazone in microsome extracts from the same livers. A probe drug such as chlorzoxazone is one metabolized almost exclusively by a single CYP isoform, in this case CYP2E1. We found that intrinsic clearance of chlorzoxazone correlated -0.3 with DNA methylation levels and 0.5 with H3K9ac levels [7]. This demonstrated that age-related epigenetic changes are significantly associated with rate of drug metabolism for CYP2E1. Importantly, chronological age of the subjects was not significantly correlated with rate of chlorzoxazone metabolism whereas the epigenetic correlations were relatively large. This suggests that epigenetic biomarkers in aging may serve as better predictors of drug metabolism than chronological age itself.

This initial study indicated the potential importance of age-related epigenetic variation on drug metabolism but there are several limitations of our work thus far. First, we only surveyed a subset of the possible epigenetic marks (5mC, H3K9ac and H3K27ac) at a single gene. A deeper understanding of genome-wide age-related changes for the full spectrum of epigenetic modifications and their effects on chromatin conformation in the liver is needed. Furthermore, while mice provide a powerful model of aging with limited environmental variation, there are significant genetic divergences between rodent and human xenobiotic metabolizing genes. While human *CYP2E1* has a mouse ortholog, some of the most important human xenobiotic metabolizing genes such as *CYP3A4* do not. This implies that studies in post-mortem human tissue may be needed to capture the most important genes. Finally, considering the translation of these findings to viable biomarkers, we also need to understand the degree to which age-related epigenetic changes in central organs involved in drug metabolism such as the liver and kidney reflect those of more accessible, peripheral tissues such as blood or fibroblasts.

In general, the field of pharmacoepigenetics is gaining traction as more research is carried out on epigenetic biomarkers of drug response in many disease areas [6]. Our recent work, although limited in scope, suggests that epigenetic changes in normal aging can have significant impact on drug metabolism.

## References

[1] Shehab N, et al. JAMA. 2016; 316:2115–25. 10.1001/jama.2016.16201 27893129 PMC6490178

[2] Routledge PA, et al. Br J Clin Pharmacol. 2004; 57:121–26. 10.1046/j.1365-2125.2003.01875.x 14748810 PMC1884428

[3] McLachlan AJ, et al. Clin Pharmacol Ther. 2009; 85:431–33. 10.1038/clpt.2009.1 19225449

[4] Gaedigk A, et al. Genet Med. 2017; 19:69–76. 10.1038/gim.2016.80 27388693 PMC5292679

[5] Horvath S. Genome Biol. 2013; 14:R115. 10.1186/gb-2013-14-10-r115 24138928 PMC4015143

[6] Kronfol MM, et al. Expert Rev Precis Med Drug Dev. 2017; 2:33–45. 10.1080/23808993.2017.1284557 29276780 PMC5737812

[7] Kronfol MM, et al. Geroscience. 2020; 42:819–32. 10.1007/s11357-020-00181-5 32221779 PMC7287002

